# Supplementary material for: Dielectric anomalies and interactions in the three-dimensional quadratic band touching Luttinger semimetal Pr2Ir2O7
Source: Nat Commun. 2017 Dec 13;8:2097. doi: 10.1038/s41467-017-02121-y (PMC5727407; doi:10.1038/s41467-017-02121-y)
Supplement: Supplementary file 1 — Supplementary Information [file 41467_2017_2121_MOESM1_ESM.pdf]

Supplemental Material

SUPPLEMENTARY NOTE 1: ESTIMATION OF FERMI ENERGY FROM ANOMALOUS HALL RESPONSE

We used dc transport data as an additional check on the Fermi energies we estimate in the paper from the Drude spectral weight. The original data appears in Supp. Ref. [1] and was taken on a similarly prepared $Pr_2Ir_2O_7$ film studied in that paper. It is well known that $Pr_2Ir_2O_7$ shows an anomalous Hall effect [2]. In general, it is assumed that anomalous Hall effect systems have a Hall resistivity that can be expressed as $\rho_{xy} = R_H|\mathbf{B}| + R_s|\mathbf{M}|$. Here, $R_H = 1/ne$ is the conventional Hall coefficient and R_s is the coefficient related to magnetization $\mathbf{M}$. Here, n is the free charge density and e is the unit charge, where a positive Hall coefficient corresponds to hole carriers. In Supplementary Figure 1a, we show the Hall resistivity as a function of magnetic field at 2 K. In Supplementary Figure 1b, we show the magnetization as a function of magnetic field at 2 K. To extract R_H, we plot in Supplementary Figure 1c the measured $\rho_{xy}/|\mathbf{B}|$ as a function of $|\mathbf{M}|/|\mathbf{B}|$.

In the region of high $|\mathbf{M}|/|\mathbf{B}|$, the curve is very non-linear, however at low $|\mathbf{M}|/|\mathbf{B}|$ a y-intercept can be defined that gives an R_H of 3.6 $\mu\Omega{\cdot}\text{cmT}^{-1}$. Using the expression for R_H we determine a free charge density $n = 1.7 \times 10^{20}$ cm^{-3}. Using the usual expressions for density and Fermi wavevector for a system with quadratic dispersion, we find $k_F = 0.17$ Å^{-1} and a Fermi energy of around 17.5 meV, which given the uncertainties in the y-intercept is very close to the values determined from the spectral weight of the THz optical conductivity.

SUPPLEMENTARY NOTE 2: CALCULATION OF $\varepsilon_{QBT}/\epsilon_0$ OF $Pr_2Ir_2O_7$ THIN FILMS FROM RPA

The dielectric constant in the presence of a quadratic band touching (QBT) has been calculated in the context of the random phase approximation (RPA) [3–6]. The RPA theory represents the lowest order contribution that takes interactions into account. As discussed in the main text, due to the weak role of higher order logarithmic corrections to the dielectric function, RPA remains an excellent approximation even in the presence of strong interactions. Following Ref. [5], within RPA for a quadratic band system with identical masses for valence and conduction bands m^*, in the limit at zero temperature, and a finite E_F, the imaginary part of the dielectric function is

$$\varepsilon'' = \epsilon_0 \sqrt{\frac{m^* e^4}{\varepsilon_\infty^2 \hbar^3 \omega}}\,\Theta(\hbar\omega - 2E_\text{F}). \tag{1}$$

Here we have modified the expression of Ref. [5] to allow for a finite background dielectric constant ε_∞. Θ is a step function that accounts for the fact that at frequencies below $2E_\text{F}$ absorptions are not possible due to Pauli blocking.

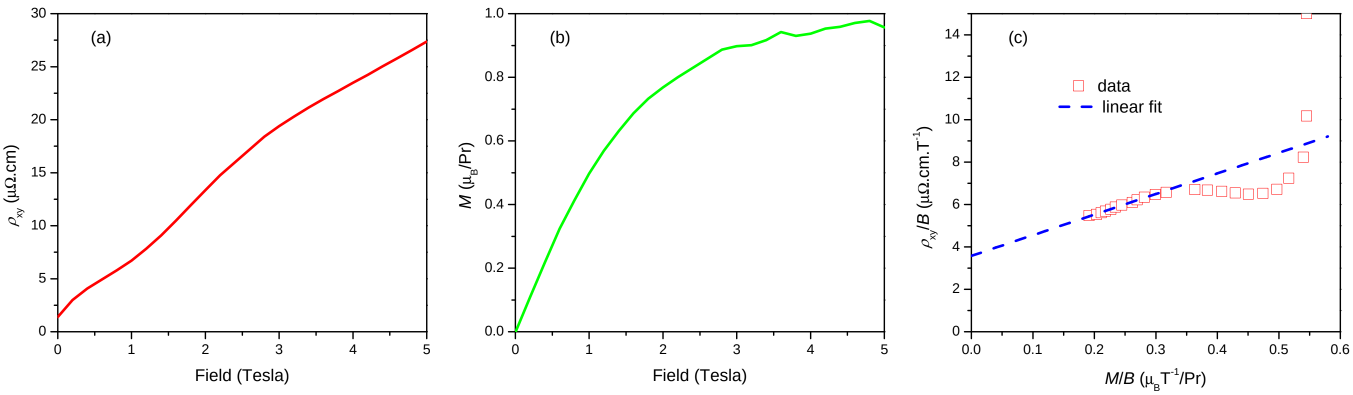

[!H]

Supplementary Figure 1: | **DC Transport measurements and anomalous Hall effect.** (a) Hall resistivity as a function of magnetic field at 2 K. (b) Magnetization as a function of a (111) directed magnetic field at 2 K. (c) $\rho_{xy}/|\mathbf{B}|$ as a function of $|\mathbf{M}|/|\mathbf{B}|$ at 2 K. The dash line shows a linear fit of the data. The y-intercept of the fit is the Hall coefficient R_H we wish to extract as discussed in the text.

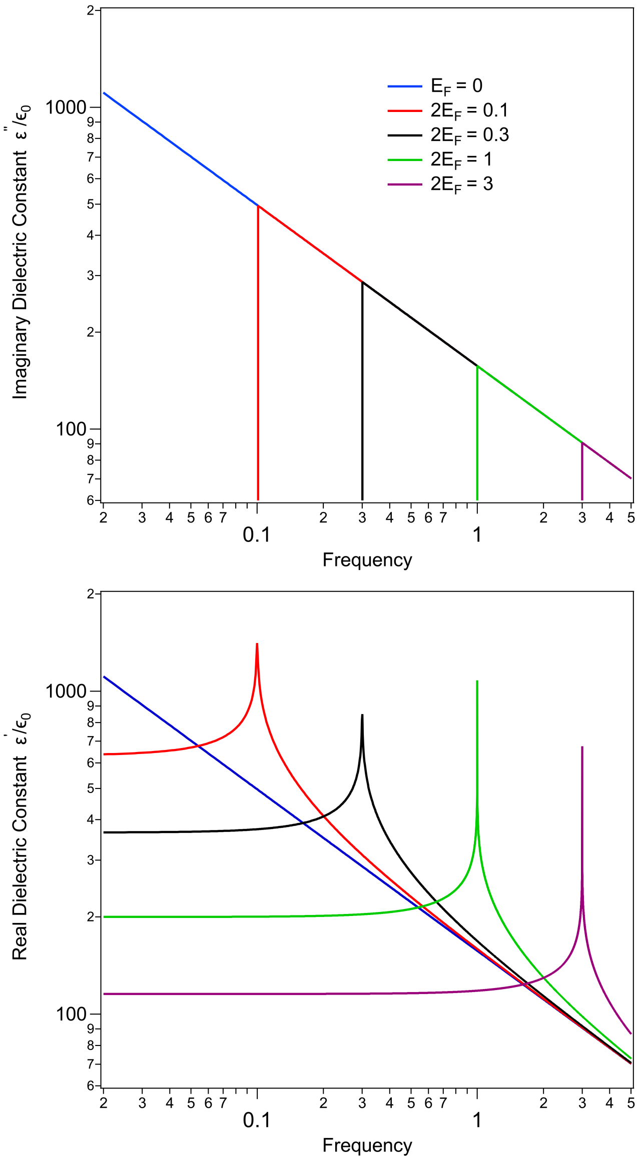

Supplementary Figure 2: | **Calculated dielectric constant for a quadratic band touching system.** Imaginary and real parts of the dielectric constant calculated from the expressions given in Eqs. 1 and 2. The calculation was done within the random phase approximation and with different representative values of the Fermi energy.

Corrections to account for unequal valence and conduction band masses are easily included [5]. The real part of the dielectric function can be found from a Kramers-Kroning transformation of Eq. 1. Again due to Pauli blocking, finite E_{F} cuts off the divergence of virtual excitations at an energy $2E_{\mathrm{F}}$ that determines ε' at low ω. Due to the sharp cut-off in ε'', ε' is logarithmically divergent at $2E_{\mathrm{F}}$. The real part of the dielectric constant is then

$$\varepsilon' = \epsilon_0 \frac{2}{\pi}\sqrt{\frac{m^* e^4}{\varepsilon_\infty^2 \hbar^3 \omega}}\left[\frac{\pi}{2} - \tan^{-1}\left(\sqrt{\frac{2E_{\mathrm{F}}}{\hbar\omega}}\right) + \frac{1}{2}\ln\left|\frac{1+\sqrt{\frac{2E_{\mathrm{F}}}{\hbar\omega}}}{1-\sqrt{\frac{2E_{\mathrm{F}}}{\hbar\omega}}}\right|\right]. \tag{2}$$

We plot these functions for ε'' and ε' in Supplementary Figure 2. Among other things, these response functions are notable in that $\varepsilon'' = \varepsilon'$ for frequencies well above the $2E_{\mathrm{F}}$ cut-off. Although the form of ε' is non-trivial near $2E_{\mathrm{F}}$, a particularly simple form exists at low frequencies. Expansion of Eq. 2 using $\tan^{-1}(\frac{1}{x}) = \frac{\pi}{2} - \tan(x) \approx \frac{\pi}{2} - x$ for $x \ll 1$ shows that the term in brackets approaches $2\sqrt{\frac{\hbar\omega}{2E_{\mathrm{F}}}}$ for $\hbar\omega \ll 2E_{\mathrm{F}}$. Therefore in the low frequency limit the dielectric constant is $4/\pi$ times the value that it has at the cutoff frequency in the cutoff-free expression (where the

last two terms in the brackets are zero). Therefore the low frequency limit can be expressed with the substitution into the cutoff-free expression of $\hbar\omega \rightarrow \frac{\pi^2}{16} 2E_{\mathrm{F}}$, similar to the case discussed in the main text for when the valence and conduction band masses are not equal. Using the expression for the exciton energy ($E_0 = \frac{m^* e^4}{64\pi^2 \varepsilon_\infty^2 \hbar^2}$) one can express the real part of the dielectric constant at low frequency as $\varepsilon' = 16\sqrt{2}\epsilon_0 \sqrt{\frac{E_0}{E_{\mathrm{F}}}}$.

[1] Ohtsuki, T. *et al.* Magnetotransport properties of pyroclore iridate $Pr_2Ir_2O_7$ epitaxial thin films. *Preprint on arXiv* (2017).

[2] Machida, Y. *et al.* Unconventional Anomalous Hall Effect Enhanced by a Noncoplanar Spin Texture in the Frustrated Kondo Lattice $Pr_2Ir_2O_7$. *Phys. Rev. Lett.* **98**, 057203 (2007).

[3] Liu, L. & Brust, D. Dielectric Singularity of α-Sn. *Phys. Rev.* **173**, 777–780 (1968).

[4] Broerman, J. G. Temperature Dependence of the Static Dielectric Constant of a Symmetry-Induced Zero-Gap Semiconductor. *Phys. Rev. Lett.* **25**, 1658–1660 (1970).

[5] Broerman, J. Random-Phase-Approximation Dielectric Function of α-Sn in the Far Infrared. *Physical Review B* **5**, 397–408 (1972).

[6] Grynberg, M., Le Toullec, R. & Balkanski, M. Dielectric function in HgTe between 8 and 300° K. *Phys. Rev. B.* **9**, 517–526 (1974).
